# Gender-Stereotyping and Cognitive Sex Differences in Mixed- and Same-Sex Groups

**DOI:** 10.1007/s10508-014-0311-5

**Published:** 2014-06-13

**Authors:** Marco Hirnstein, Lisa Coloma Andrews, Markus Hausmann

**Affiliations:** 1 Department of Biological and Medical Psychology, University of Bergen, Jonas Lies vei 91, 5009 Bergen, Norway; 2 Institute for Stroke and Dementia Research, Ludwig Maximilian University, Munich, Germany; 3 Department of Psychology, Durham University, Durham, UK

**Keywords:** Gender stereotypes, Mental rotation, Verbal fluency, Group sex composition, Stereotype threat

## Abstract

Sex differences in specific cognitive abilities are well documented, but the biological, psychological, and sociocultural interactions that may underlie these differences are largely unknown. We examined within a biopsychosocial approach how gender stereotypes affect cognitive sex differences when adult participants were tested in mixed- or same-sex groups. A total of 136 participants (70 women) were allocated to either mixed- or same-sex groups and completed a battery of sex-sensitive cognitive tests (i.e., mental rotation, verbal fluency, perceptual speed) after gender stereotypes or gender-neutral stereotypes (control) were activated. To study the potential role of testosterone as a mediator for group sex composition and stereotype boost/threat effects, saliva samples were taken before the stereotype manipulation and after cognitive testing. The results showed the typical male and female advantages in mental rotation and verbal fluency, respectively. In general, men and women who were tested in mixed-sex groups and whose gender stereotypes had not been activated performed best. Moreover, a stereotype threat effect emerged in verbal fluency with reduced performance in gender stereotyped men but not women. Testosterone levels did not mediate the effects of group sex composition and stereotype threat nor did we find any relationship between testosterone and cognitive performance in men and women. Taken together, the findings suggest that an interaction of gender stereotyping and group sex composition affects the performance of men and women in sex-sensitive cognitive tasks. Mixed-sex settings can, in fact, increase cognitive performance as long as gender-stereotyping is prevented.

## Introduction

Sex differences in specific cognitive abilities are well documented (Halpern, 2000; Kimura, 2000). Although cognitive performances of both sexes overlap to a large extent, several meta-analyses demonstrate that, on average, men outperform women in certain spatial tasks (Linn & Peterson, 1985; Masters & Sanders, 1993; Voyer, Voyer, & Bryden, 1995), particularly in mental rotation (Peters et al., 1995; Vandenberg & Kuse, 1978). Women, on the other hand, excel in specific aspects of verbal abilities, such as verbal fluency or verbal memory (Hyde & Linn, 1988; McGlone, 1980) as well as perceptual speed (Feingold, 1992; Hedges & Nowell, 1995). The origins of these cognitive sex differences are still not fully understood, but they appear to arise from a complex interaction of biological, social, and psychological factors (Halpern, 2000).

An important sociocultural factor that affects cognitive sex differences are gender stereotypes. Activating gender stereotypes can have adverse or beneficial effects on cognitive performance in men and women depending on whether participants appraise the testing situation as threatening or challenging. The so-called stereotype threat effect refers to the phenomenon that participants perform poorly on a task because they are afraid of confirming negative stereotypes about their group’s alleged inferior abilities (Steele & Aronson, 1995). For instance, when women were told that a math test yields sex differences they performed more poorly than men while there was no significant sex difference when the test was described as gender neutral (Spencer, Steele, & Quinn, 1999). Likewise, women scored lower in mental rotation tests when they were told that men generally perform better in spatial abilities (Moè, 2012; Moè & Pazzaglia, 2006; Wraga, Helt, Jacobs, & Sullivan, 2007). Conversely, stereotype boost occurs when activation of gender stereotypes enhances cognitive performance (e.g., Shih, Pittinsky, & Ambady, 1999; Walton & Cohen, 2003), either by inducing positive stereotypes about the in-group (“You are good at this task”) or negative stereotypes about an out-group (“They are bad at this task”). For example, women showed enhanced performance in mental rotation tests when they were told these tests measure perspective-taking abilities in which they are superior to men (Heil, Jansen, Quaiser-Pohl, & Neuburger, 2012; Moè, 2009; Wraga, Duncan, Jacobs, Helt, & Church, 2006; Wraga et al., 2007). Stereotype boost and stereotype threat can thus increase or decrease sex differences in sex-sensitive cognitive tasks.

An important biological factor for cognitive sex differences are sex hormones, although the exact nature of the relationship between sex hormones and cognition is still unclear. Testosterone (T) in particular has been shown to have organizational effects during prenatal neural development with consequences for cognitive abilities later in life (e.g., Grimshaw, Sitarenios, & Finegan, 1995) but the present study focused on activational effects that occur throughout life by its non-genomic, direct neuromodulatory effects on brain functions and cognitive abilities. For example, it has been reported that in men higher T levels determined during the time of testing were associated with higher mental rotation scores (Hooven, Chabris, Ellison, & Kosslyn, 2004; Silverman, Kastuk, Choi, & Phillips, 1999). Moreover, administering androgens to female-to-male transsexuals can lead to enhanced performance in mental rotation (Slabbekoorn, van Goozen, Megens, Gooren, & Cohen-Kettenis, 1999; van Goozen, Cohen-Kettenis, Gooren, Frijda, & van de Poll, 1994). On the other hand, administering T to men with androgen deficiency did not improve mental rotation (Alexander et al., 1998; Liben et al., 2002) but enhanced verbal fluency (Alexander et al., 1998), showing that verbal fluency is associated with T. However, many studies did not find relationships between T and cognitive abilities (e.g., Auyeung et al., 2012; Wisniewski, Prendeville, & Dobbs, 2005; for review, see Yang, Hooven, Boynes, Gray, & Pope, 2007). Although a comprehensive review of organizational and activational effects of T is beyond the scope of the present study, it is clear that we are far from understanding the relationship between T and cognitive performance. However, despite inconsistent findings and large controversy, it seems fair to say that the conclusion made by Halpern (2000) and Kimura (2000) still holds: sex hormones, and particularly T, seem to contribute at least partly to cognitive sex differences.

While there is a high consensus that cognitive sex differences arise from interactions between biological and sociocultural factors (Halpern, 2000), surprisingly few studies have examined these interactions. A first link between T, gender stereotypes, and cognitive sex difference was suggested by Josephs, Newman, Brown, and Beer (2003). They found that women with high natural T levels (based on a median split) performed relatively poorly in a math test when they were exposed to negative stereotypes (i.e., stereotype threat) while men with high natural T levels excelled when faced with positive stereotypes (i.e., stereotype boost). No significant gender stereotype effects were observed in both men and women with low T levels. Josephs et al. concluded that T acts as a moderator variable in stereotype boost/threat situations: men with high T levels view a math test as a chance to enhance their social status and thus achieve high scores when stereotyped. However, women with high T levels view a math test as a threat to their social status and, consequently, achieve low scores when confronted with gender stereotypes.

Hausmann, Schoofs, Rosenthal, and Jordan (2009) further elaborated the link between T and gender stereotypes and proposed that T might be a mediating factor between stereotype boost and cognitive sex differences. Their participants completed a series of sex-sensitive cognitive tasks, including mental rotation, verbal fluency, and perceptual speed. Half of the participants completed a questionnaire prior to cognitive testing which aimed to activate participants’ gender stereotypes while the other half completed a gender-neutral questionnaire (control). The well-documented male advantage in mental rotation only emerged in gender-stereotyped participants. The mental rotation performance was significantly higher in gender-stereotyped men, as compared to male controls, indicating a stereotype boost whereas the performance was lower in gender-stereotyped women than female controls. Moreover, T levels were found to be 60 % higher in gender-stereotyped men than those of men in the control condition and positively related to mental rotation performance. Hausmann et al. suggested that the gender stereotype activation resulted in a rise in T levels, which then could have modulated brain areas involved in mental rotation and enhanced mental rotation scores. However, T levels in this study were only measured once, after stereotype activation. Thus, this study did not provide direct evidence for a rise in T levels due to activation of gender stereotypes. It is important to note, that all participants were tested in mixed-sex groups. This may have additionally created a competitive environment, resulting in an increase in T levels (cf. Archer, 2006) and stereotype boost. Members of the other sex are often present during tests and exams in natural settings (e.g., in classrooms at school, job assessment centers, etc.), but little is known about how this affects cognitive performance in men and women, particularly in sex-sensitive cognitive tasks when gender stereotypes are activated. Inzlicht and Ben-Zeev (2000; see also Huguet & Regner, 2007) found that women’s performance in math tests was reduced when males were present and gender stereotypes activated. Whether similar effects of gender stereotypes in mixed-sex groups also apply to sex-sensitive spatial and verbal abilities has not yet been investigated.

The aim of the present study was to investigate whether gender stereotypes affect cognitive sex differences only in mixed-sex groups or whether they also apply to same-sex settings which may have important implications for the ongoing debate on co-education. To this end, participants completed five tasks (two mental rotation tests, two verbal fluency tests, and one perceptual speed test) that consistently show sex differences in cognitive abilities. Half of the participants completed the tasks after gender stereotypes had been activated, the other half served as control. In each of these conditions, half of the participants were tested in same-sex groups and the other half in mixed-sex groups. We hypothesized that (1) the activation of gender stereotypes increases sex differences in all tasks (Sex by Condition interaction) by either enhancing performance of positively stereotyped participants (e.g., females’ performance in verbal fluency) or by reducing performance of negatively stereotyped participants (e.g., males’ performance in verbal fluency). We further hypothesized that (2) cognitive sex differences will be largest if gender-stereotypes are activated in a mixed-sex test setting (Sex by Condition by Group Sex Composition interaction). By measuring participants’ T levels twice, once before implementing gender stereotypes (or gender neutral stereotypes) and once after cognitive testing, the present study allowed us to examine changes in T levels as a consequence of the competitive testing situation related to gender stereotypes and group sex composition. Based on Hausmann et al. (2009) and Josephs et al. (2003) and the general assumption that T levels are related to cognitive performance, we hypothesized (3) that if cognitive performance is enhanced after gender stereotype activation, there will be a rise in T levels, particularly in mixed-sex groups. In contrast, stereotype threat might be associated with a T drop. In addition, we investigated (4) whether T levels in general were correlated with cognitive performance and whether cognitive performance was correlated with the magnitude of the corresponding gender stereotype. That is, the more strongly females are convinced that females in general excel in verbal fluency, the higher their verbal fluency performance (and the more strongly males are convinced, the lower their performance).

## Method

### Participants

In total, 148 adults (78 women, 70 men), recruited at the Department of Psychology, Ruhr-University Bochum, Germany, were tested. Twelve participants were excluded because of neurological conditions, cognitive performance of more than two SDs below average in all five cognitive tests, or missing data. The mean age for the remaining 70 women was 24.40 years (*SD* = 4.9) and 25.56 years (*SD* = 4.3) for the remaining 66 men. The number of participants across all conditions are shown in Table 1. Participants were tested in either mixed- or same-sex groups of 6–10 people. Participants in mixed-sex groups were always invited so that there was an equal number of men and women. Due to late cancellations, etc., occasionally minor imbalances in the sex ratio occurred. All group members were tested—all in the same condition (i.e., either gender-stereotyped or control). Interactions between participants during the experiment were kept to a minimum as they were either occupied with questionnaires or timed cognitive tasks. Moreover, participants were seated with enough space between them so that they could not copy from each other. All participants were naïve to the study’s hypotheses.

**Table 1:** Items of the gender stereotype questionnaire and mean percentage probability estimates for being male after cognitive testing

Items	Gender stereotypes activated				Control			
	Males		Females		Males		Females	
	Same-sex (*n* = 13)	Mixed-sex (*n* = 19)	Same-sex (*n* = 17)	Mixed-sex (*n* = 17)	Same-sex (*n* = 16)	Mixed-sex (*n* = 18)	Same-sex (*n* = 20)	Mixed-sex (*n* = 16)
“You are going to meet a person whom you have never met before. What is the probability that this person is male given that this person …”								
(1) has problems recognizing a complicated drawing when he/she sees it upside-down	40.1	44.7	42.6	41.2	40.9	42.8	45.3	37.5*
(2) can imagine common objects from different perspectives	48.8	58.4	54.1	58.2	56.9	61.4*	52.8	60.3*
(3) can easily remember names of guests on a party	37.7*	43.7	32.9*	38.2*	36.9*	34.4*	35.3*	36.9*
(4) often makes spelling mistakes	56.9	55.5	57.4	57.6*	52.8	58.9*	60.0*	56.6*
(5) can draw a map of the area where he/she lives	62.3	62.1*	56.5	56.2	61.6*	66.7*	60.8*	60.3*
(6) is bad at reading street maps	33.1*	35.8*	35.9*	35.9*	41.9	37.8	45.3	36.3*
(7) has problems summarizing books or movies in a short and clear manner	45.4	47.6	59.7	54.1	50.6	58.1	48.0	59.4*
(8) does not use landmarks for orientation^a^	41.5	39.7*	54.7	49.7	46.9	41.7	50.0	57.8
(9) can speak three different languages fluently	35.4*	40.3*	36.5*	33.5*	37.3*	33.6*	36.0*	41.6*
(10) can imagine abstract objects and rotate them mentally in all directions	60.4	62.9*	68.2*	67.6*	58.8*	67.2*	65.0*	67.8*
(11) often forgets where common objects like keys were put	48.5	48.7	56.5	55.3	50.9	55.0	53.5	55.0
(12) can generate many words beginning with the same letter within 1 min	40.8*	41.3*	36.5*	37.6*	43.1*	38.3*	43.3*	39.1*
(13) finds it difficult to imagine common objects and rotate them mentally	40.4	44.7	38.2	43.8	41.1*	41.7	42.3*	38.1*
(14) remembers a route based on left–right turnoffs	62.3*	52.4	61.7	64.4*	56.9	54.4	54.3	54.1
(15) cannot think of many synonyms for a specific term^b^	51.5	55.3	49.4	54.1	55.6	57.5	49.8	63.1*
(16) can easily summarize the essentials from a newspaper article	49.2	46.8	46.2	48.2	45.1	46.4	51.8	42.5*

* Mean probability differs significantly (*p* < .01) from 50 according to one-sample *t*-test indicating a gender stereotype

^a^Main effect of Sex: men believed such a person was more likely to be female (*M* = 42.5 ± SE = 2.0) while women believed such a person was more likely to be male (57.5 ± 1.5)

^b^Main effect of Group Sex Composition: Participants in mixed-sex groups (57.5 ± 1.5) believed more strongly that such a person was male than participants in same-sex groups (51.6 ± 1.5)

### Procedure and Measures

#### Gender Stereotype Questionnaire

Before participants completed the cognitive tests, they completed either a gender stereotype or gender-neutral stereotype questionnaire, which were identical to Hausmann et al. (2009; adapted from Halpern & Tan, 2001). In both groups, participants were asked to imagine that they were about to meet a person whom they had never met before. In the experimental group, participants then estimated the percentage probability that this person was male or female (both estimates should sum up to 100) based on the fact that this person, for example, “speaks three foreign languages fluently.” The gender stereotype questionnaire comprised 16 items with statements about cognitive abilities (see Table 1) that relate to the spatial and verbal skills tested in the present study. The gender stereotype questionnaire thus did not only induce gender stereotypes, but also allowed them to be quantified. Participants in the control group received a gender-neutral questionnaire with the identical 16 items from the gender stereotype questionnaire, but they estimated the probability that a person whom they had never met before was “Northern American” or “European.”

After cognitive testing, participants were asked to complete the questionnaire a second time, but now participants in the control group also received the gender stereotype version. This procedure allowed to investigate whether (1) cognitive testing led to changes in gender stereotypes in the experimental group and (2) participants in the control group held similar gender stereotypes as the experimental group.

#### Cognitive Tests

The Redrawn Vandenberg and Kuse Mental Rotation Test (Version A) (Peters, 1995), henceforth referred to as *MRT*-*3D*, was originally developed by Vandenberg and Kuse (1978) and consists of drawings of 3-dimensional cube figures (Shepard & Metzler, 1971). Each item in the MRT-3D comprises five of these figures: one target and four sample figures. Two of these four sample figures are identical but rotated versions of the target figure. One point is given if both identical figures are correctly identified. The MRT-3D contains two parts with 12 items each, so the maximum score is 24. For each part, there was a time limit of 3 min (for more details, see Peters et al., 1995).

The Mirror Pictures (MP-2D) is a subtest of the WILDE-Intelligenz-Test (Jäger & Althoff, 1994). Similar to the MRT-3D, participants were presented drawings of five 2-dimensional symbols. Four of them were identical but rotated and one was mirror-inverted. If the latter one was identified correctly, participants received one point. The MP-2D consists of 24 items and participants had a time limit of 3 min. Meta-analyses show that the sex difference favoring males is about *d* = 0.6 in two-dimensional mental rotation tests such as the MP-2D and up to one SD in the MRT-3D (Linn & Petersen, 1985; Masters & Sanders, 1993; Voyer et al., 1995).

In the Word Fluency Test (WF), a subtest of the Leistungsprüfsystem (Horn, 1962), participants were asked to write down as many nouns (excluding proper nouns, variations, and repetitions) as possible starting with either the letter “L” or “P.” They had 1 min per letter and one point was given for each correct noun.

In the 4-Word Sentences Test (4W), a subtest of the Verbaler–Kreativitäts-Test (Schoppe, 1975), participants were presented with two four letter arrays (T-G-F-U and B-H-K-N) on a sheet of paper. They had 2.5 min per array to write down as many sentences as possible such that each word in the sentence started with one of the letters. The sentences did not need to be meaningful but they had to be grammatically correct. One point was given per correct sentence. Verbal fluency tests typically reveal small sex differences of about *d* = .20 (Hyde & Linn, 1988).

The Perceptual Speed Test (PS) is another subtest of the WILDE-Intelligenz-Test (Jäger & Althoff, 1994). Participants were shown drawings of three faces. Two of them were identical; one drawing differed in a detail (such as a missing eyebrow) and had to be identified. The test contains 42 items. Every correctly identified face received one point and there was a time limit of 3 min. Effect sizes in perceptual speed tasks as the PS reveal small effect sizes between *d* = .21 and *d* = .43 favoring women (Hedges & Nowell, 1995).

#### Testosterone Assays

To investigate participants’ T levels, saliva samples were collected twice, once before the gender stereotype manipulation and once directly after data collection, that is, when all cognitive tests and questionnaires were completed. The experimental session took about 60–80 min. During such a period of time, changes in T levels have been demonstrated (e.g., Carré & Putnam, 2010). Saliva samples were stored at −22 °C until all participants had completed the experiment. Free T levels were determined with Chemiluminescent Immunoassay (LIA) by an independent professional hormone laboratory, with commercially available LIA kits. The intra-assay coefficients of variations (CVs) were 4.0 and 8.1 %, and the inter-assay CVs were 7.4 and 11.7 % for high and low T levels, respectively. From participants who agreed to provide saliva samples, only samples not contaminated with blood (e.g., from gum bleeding) were analyzed. According to these criteria, four participants (one woman, three men) were excluded from analyses that included T levels, but they remained in all other analyses.

## Results

### Gender Stereotypes

The dependent variable of the gender stereotype questionnaire was the estimated percentage probability of “being male.” Values above 50 indicated a participant’s belief that a person who, for example, “speaks three foreign languages fluently,” was more likely to be male while values below 50 indicated that such a person was more likely to be female. The probability estimates for all 16 items in the gender stereotype version (after cognitive testing) are shown in Table 1. One-sample *t*-tests (test value 50 = equal probability for being male or female) were computed in each group to examine whether biases towards one gender exist. As can be seen in Table 1, females were generally associated with higher verbal and males with higher spatial skills.

To investigate whether the two experimental groups showed similar gender stereotypes, probability means were subjected to a 2 (Sex) × 2 (Condition: Gender-Stereotyped vs. Control) × 2 (Group Sex Composition: Same- vs. Mixed-Sex) analysis of variance (ANOVA). Only a main effect of Sex, *F*(1, 128) = 14.31, *p* < .001, for Item 8 and a main effect of Group Sex Composition, *F*(1, 128) = 7.68, *p* = .006, for Item 15 emerged (see Table 1). No further main effects or interactions were significant across all 16 items (all *F*s ≤ 6.80).

In addition, we examined whether gender stereotypes changed from before to after cognitive testing by subjecting probability estimates for all 16 items in the gender-stereotyped group to a 2 (Sex) × 2 (Group: Same- vs. Mixed-Sex) × 2 (Pre-/Post cognitive testing) ANOVA. A main effect of Pre-/Post cognitive testing was found for Item 5, *F*(1, 62) = 14.13, *p* < .001. Before stereotype manipulation (64.2 ± 2.0), participants were significantly more convinced that a person who “can draw a map of the area where he/she lives” was more likely to be male than after testing (59.3 ± 1.8). No further main effects or interactions involving Pre-/Post cognitive testing were significant (all *F*s ≤ 5.01). To avoid alpha-error inflation, the significance level in all analyses was set to 1 %.

Overall, the analyses of individual questionnaire items indicated pronounced gender stereotypes, which were relatively stable over the time of testing and differed only marginally between groups and conditions.

### Cognitive Test Performance

In order to test Hypotheses 1 and 2 according to which sex differences were expected to be exacerbated when gender stereotypes had been activated in mixed-sex groups, test scores of the MRT-3D, MP-2D, WF, 4W, and PS were subjected to 2 (Sex) × 2 (Condition: Gender-Stereotyped vs. Control) × 2 (Group Sex Composition: Same- vs. Mixed-Sex) ANOVAs. The alpha-level was set at .05, post hoc *t*-tests were carried out with Bonferroni adjustment, and effect sizes are given as the proportion of variance accounted for (partial *η* ^2^). For sex differences, Cohen’s *d* is additionally provided to facilitate comparison with previous studies. Means and SEs are shown in Table 2.

**Table 2:** Mean cognitive performance (±SE) in mental rotation (MRT-3D and MP-2D), verbal fluency (4W and WF), and perceptual speed (PS) for men and women across condition (gender stereotype, control) and group sex composition (same- or mixed sex groups)

Tasks	Gender stereotypes activated				Control			
	Same-sex group		Mixed-sex group		Same-sex group		Mixed-sex group	
	Males	Females	Males	Females	Males	Females	Males	Females
Male-favoring								
MRT-3D (Max 24)	12.00 ± 1.31	9.88 ± 1.15	11.89 ± 1.08	9.24 ± 1.15	11.75 ± 1.18	9.00 ± 1.06	12.61 ± 1.11	9.31 ± 1.18
MP-2D (Max 24)	12.00 ± 1.40	14.59 ± 1.22	15.58 ± 1.15	13.53 ± 1.22	16.38 ± 1.26	15.10 ± 1.12	17.00 ± 1.18	14.75 ± 1.26
Female-favoring								
WF (no Max)	20.00 ± 1.76	24.12 ± 1.54	19.21 ± 1.46	24.71 ± 1.54	19.75 ± 1.60	21.60 ± 1.42	24.28 ± 1.50	26.19 ± 1.59
4W (no Max)	7.15 ± .88	9.94 ± .77	6.79 ± .73	8.94 ± .77	8.56 ± .79	8.15 ± .71	10.61 ± .75	10.25 ± .79
PS (Max 42)	16.08 ± 1.33	17.12 ± 1.17	15.63 ± 1.10	16.71 ± 1.17	15.81 ± 1.20	18.90 ± 1.07	22.44 ± 1.13	20.19 ± 1.20

#### Mental Rotation

For the MRT-3D, the ANOVA revealed a significant main effect of Sex with higher scores for men than women, *F*(1, 128) = 10.97, *p* = .001, *η* ^2^ = .08, *d* = 0.57. There were no other significant main effects or interactions (all *F*s ≤ 1).

In the MP-2D, the main effect of Condition was significant, *F*(1, 128) = 4.70, *p* = .032, *η* ^2^ = .04, indicating that participants in the control condition achieved higher scores than those in the gender-stereotyped condition. No further main effects or interactions were significant (all *F*s ≤ 2.61).

#### Verbal Fluency

**Fig. 1:**
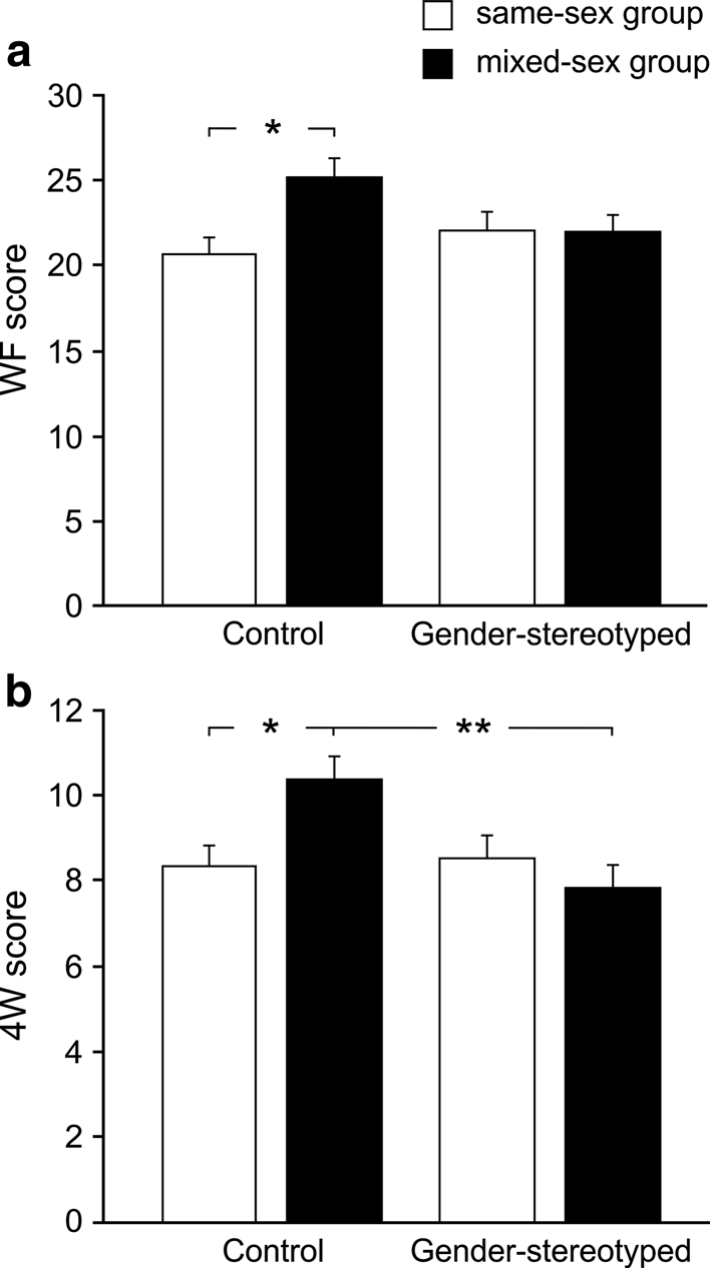
Mean verbal fluency scores (+SE) across condition and group sex composition in WF (**a**) and 4W (**b**) show enhanced performance in non-stereotyped mixed-sex settings. *p < .05, **p < .01

For WF, the ANOVA revealed a main effect of Sex, *F*(1, 128) = 9.25, *p* = .003, *η* ^2^ = .07, *d* = 0.53, indicating that women obtained higher verbal fluency scores than men. Moreover, there was an interaction between Condition and Group Sex Composition, *F*(1, 128) = 4.49, *p* = .036, *η* ^2^ = .03. Controls in mixed-sex groups achieved the highest scores (see Fig. 1a), differing significantly from controls in same-sex groups (*p* = .027). No other post hoc *t* test reached significance (*p*s > .05). The higher performance of controls in mixed-sex groups led to a significant main effect of Group Sex Composition, *F*(1, 128) = 4.11, *p* = .045, *η* ^2^ = .03, with participants in mixed-sex groups (23.60 ± 0.76) outperforming participants in same-sex groups (21.37 ± 0.79). No other main effect or interactions were significant (all *F*s ≤ 1.77).

**Fig. 2:**
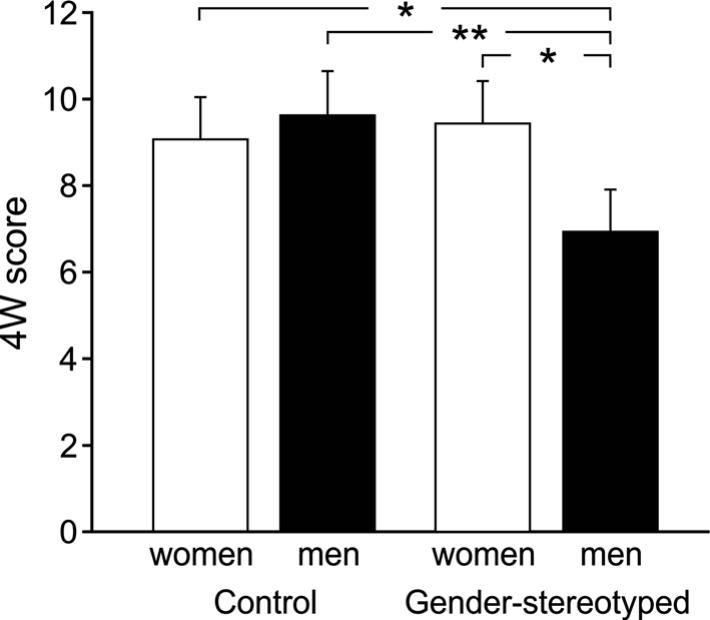
Mean verbal fluency scores (+SE) across condition and sex in 4W show typical stereotype threat effect. *p < .05, **p < .01

For 4W, there was a Sex × Condition interaction, *F*(1, 128) = 6.77, *p* = .011, *η* ^2^ = .05. As predicted in Hypothesis 1, there was no significant difference between men (9.65 ± 0.63) and women (9.08 ± 0.55) in the control condition (*p* > .05), but verbal fluency was significantly lower in men (6.94 ± 0.48) than women (9.44 ± 0.53) in the gender stereotype condition (*p* = .010, *d* = 0.87). Gender-stereotyped men also had significantly lower scores than non-stereotyped men (*p* = .004) and non-stereotyped women (*p* = .038) (see Fig. 2) (all other *p*s > .05). As in WF, a significant interaction between Condition and Group Sex Composition emerged, *F*(1, 128) = 6.30, *p* = .013, *η* ^2^ = .05, with controls in mixed-sex groups achieving the highest 4W score, see Fig. 1b. They outperformed controls in same-sex groups (*p* = .038) and gender-stereotyped participants in mixed-sex groups (*p* = .004, all other *p*s > .05). Furthermore, the main effect of Condition was significant, *F*(1, 128) = 4.67, *p* = .033, *η* ^2^ = .04, with lower scores in the gender stereotype condition (8.21 ± 0.40) than in the control condition (9.39 ± 0.38), and a trend towards higher scores in women was observed, *F*(1, 128) = 3.60, *p* = .06, *η* ^2^ = .03, *d* = 0.33. No other main effect or interaction reached significance (all *F*s ≤ 1.61).

#### Perceptual Speed

For PS, the ANOVA revealed a significant interaction between Condition and Group Sex Composition, *F*(1, 128) = 6.98, *p* = .009, *η* ^2^ = .05. As in verbal fluency, controls in mixed-sex groups obtained the highest score. They significantly outperformed controls in same-sex groups (*p* = .006) as well as gender-stereotyped participants in same-sex (*p* < .001) and mixed-sex groups (*p* < .001). Paired comparisons between the three same-sex groups did not reach significance (all *p*s > .05). As a result of the higher performance of controls in mixed-sex groups, there was a significant main effect of Condition, *F*(1, 128) = 12.65, *p* = .001, *η* ^2^ = .09, and Group Sex Composition, *F*(1, 128) = 4.52, *p* = .035, *η* ^2^ = .03, with higher performance in controls and mixed-sex groups compared to gender-stereotyped and same-sex participants, respectively. There were no other main effects or interactions (all *F*s ≤ 2.62).

Despite a relatively large sample size (*N* = 136), the predicted 3-way interaction between Condition, Group Sex Composition, and Sex (Hypothesis 2) did not approach significance in any cognitive test. A power analysis (G*Power 3.1.2) revealed a power of .82 (based on *α* = .05 and a medium effect size *d* = 0.50) to detect a three-way interaction.

### Testosterone Levels

To test whether T changes occurred with respect to stereotype boost or stereotype threat (Hypothesis 3), T levels from saliva samples were subjected to a (Sex) × 2 (Condition: Gender-Stereotyped vs. Control) × 2 (Group Sex Composition: Same- vs. Mixed-Sex) × 2 (Pre-/Post stereotype manipulation) mixed-design ANOVA. As expected, a main effect of Sex, *F*(1, 124) = 240.23, *p* < .001, *η* ^2^ = .66, revealed significantly higher T levels in men (107.20 ± 3.77 pg/mL) than women (26.51 ± 3.59 pg/mL). No other effects were significant (all *F*s ≤ 3.11).

### Relationship Between Gender Stereotypes, Testosterone and Cognitive Performance

Finally, we investigated whether there was a general association between T, gender stereotypes, and cognitive performance (Hypothesis 4). Multiple linear regressions were computed for each cognitive test (MRT-3D, MP-2D, 4W, WF, and PS) with the specific test score as the dependent variable and T levels (after the experiment) as well as gender stereotypes as predictors.

To examine the association of gender stereotypes and cognitive performance, probability estimates of Item 10 (*can imagine abstract objects and rotate them mentally in all direction*) and Item 12 (*can generate many words beginning with the same letter within 1* *min*) from the gender stereotype questionnaire that all participants completed at the end of the experiment were used. We focused on those two items because Item 10 and Item 12 directly relate to the mental rotation (MRT-3D and MP-2D) and verbal fluency (WF and 4W) tasks, respectively. Specifically, Item 10 was used as a predictor for MRT-3D and MP-2D, while Item 12 was used as a predictor for WF and 4W. No gender stereotype item was used for PS. Multiple regressions were conducted separately for men and women because it has previously been suggested that the relationship between T and cognitive performance is sex-specific (e.g., Kimura, 2000). Given that T levels are highly correlated with sex, this procedure additionally avoids multicollinearity.

In men, none of the cognitive tasks showed a significant model (all *F*s ≤ 2.71). Only in MRT-3D, gender stereotype was a significant predictor (*β* = .26, *p* = .041), indicating a positive correlation between MRT-3D score and the probability that somebody who was good at mental rotation was male (i.e., the better men performed on the MRT-3D the stronger was their gender stereotype that males excel in mental rotation). T levels were not significantly correlated with cognitive performance (all *β*s ≤ .16). In women, a significant model only emerged in 4W, *F*(2, 66) = 4.73, *p* = .012, accounting for 13 % of variance (all other *F*s ≤ 1.84). Only the gender stereotype significantly predicted the 4W score (*β* = −.31, *p* = .009), indicating a negative correlation between 4W scores and the probability that somebody who was good at verbal fluency was male (i.e., the better women performed on the 4W the stronger was their gender stereotype that women excel in verbal fluency). Again, T levels did not significantly predict any of the cognitive tasks scores (all *β*s ≤ .16). Since T levels before and after the experiment were highly correlated, *r* = .92, *p* < .00001, regressions did not include both predictors because of multicollinearity. The results did not change if the predictor T levels after the experiment, as reported above, was replaced by pre-test T levels.

## Discussion

### Gender Stereotypes

In order to examine the effects of gender-stereotyping on cognitive sex differences in mixed-sex and same-sex settings, it was necessary that our participants demonstrated robust gender stereotypes with respect to verbal and spatial abilities. Fortunately, the gender stereotype questionnaire revealed that participants of both sexes believed that males, rather than females, were more likely to do well on spatial tasks and that females, rather than males, were more likely to do well in verbal tasks. These findings were consistent with two previous studies which used the same gender stereotype questionnaire (Hausmann et al., 2009; Hirnstein, Freund, & Hausmann, 2012). The gender stereotypes remained stable across cognitive testing and were very similar across men and women, across participants in the control and gender stereotype condition, and across participants in mixed and same-sex groups. The observed sex differences in cognitive performance were thus unlikely to arise from differences in preexisting gender stereotypes. Finally, in accordance with Hypothesis 4, we found that men and women who performed better on mental rotation (MRT-3D) and verbal fluency (4W), respectively, also held stronger gender stereotypes regarding spatial and verbal skills. As gender stereotypes for all participants were measured after cognitive testing, this shows that cognitive performance may strengthen gender stereotypes.

### Gender Stereotypes and Cognitive Sex Differences

Overall, there was a female advantage in verbal fluency (WF: *d* = 0.53, 4W: *d* = 0.33), which was consistent with meta-analyses and comprehensive reviews on sex differences in verbal abilities (Halpern, 2000; Hyde & Linn, 1988; Kimura, 2000). In the control condition, however, men generated as many four-word sentences (4W) as women whereas men’s performance was significantly reduced when gender stereotypes were activated (see Fig. 2). This is a typical stereotype threat effect consistent with Hypothesis 1. No stereotype threat emerged in the other verbal fluency test (i.e., WF). This discrepancy between tasks can be explained by the fact that stereotype threat emerges particularly in cognitively more demanding tasks (Ben-Zeev, Fein, & Inzlicht, 2005; Keller, 2007; O’Brien & Crandall, 2003). Since 4W is considered to be more demanding than WF, because whole sentences instead of only single words need to be generated, this might explain the emergence of stereotype threat in 4W only. To our knowledge, this was the first evidence of a stereotype threat in men’s verbal abilities, which is in contrast to a previous study (Hirnstein et al., 2012) that found enhanced verbal fluency in WF and 4W in gender-stereotyped men and women. The different outcomes might be due to differences in the gender stereotype manipulation as in Hirnstein et al. participants were explicitly told that sex differences were expected, which might have induced a competitive situation leading to increased effort.

In mental rotation, the typical male advantage emerged on the MRT-3D with an effect size *d* = 0.57 in the typical range (Estes & Felkner, 2012; Voyer et al., 1995). The sex difference in MP-2D was nonsignificant and negligible in size (*d* = 0.15) and was consistent with previous findings showing that 3-dimensional objects yield stronger sex differences than 2-dimensional objects (e.g., Peters et al., 1995). Unexpectedly, the sex difference in MRT-3D was unaffected by the gender stereotype manipulation. Several studies have reported gender-related stereotype boost/threat effects in mental rotation (Moè, 2009, 2012; Moè & Pazzaglia, 2006; Wraga et al., 2006, 2007). Most importantly, we did not replicate the enhanced MRT-3D scores in gender-stereotyped men as reported in Hausmann et al. (2009) although the gender stereotype manipulation was identical and participants in both studies showed similarly pronounced gender stereotypes with respect to spatial abilities. This cannot be attributed to a failure of the stereotype manipulation, since our stereotype intervention successfully induced stereotype threat and group sex composition effects in other tasks. It is also rather unlikely that we recruited an unusual sample, since well-known sex differences in mental rotation and verbal fluency were found. The results of the present study rather suggest that it is difficult to induce stereotype threat and boost simultaneously when the test battery includes tasks favoring men and women.

In addition, and similar to Hausmann et al. (2009), the perceptual speed test neither revealed significant sex differences (but see Hedges & Nowell, 1995) nor any gender stereotype effects.

### Gender Stereotyping in Mixed- and Same-Sex Groups

Hypothesis 2 was that stereotype boost or threat effects, such as men’s reduced performance in verbal fluency, are particularly pronounced in mixed-sex groups. However, no three-way interaction emerged. Although statistical power was acceptable (*β* = .82), it cannot be ruled out that a larger sample size is needed to detect this effect. Alternatively, it is possible that gender-stereotyped men in same-sex groups simply anticipated comparison with women and therefore showed similar effects as men in mixed-sex settings. Although no three-way interaction emerged, the present findings clearly demonstrated that the impact of gender stereotype activation on cognitive performance depended upon the presence or absence of other men or women. In verbal fluency (WF, 4W) and PS, both male and female participants in mixed-sex groups, whose gender stereotypes had not been activated, achieved the highest scores. They outperformed their gender-stereotyped counterparts in mixed-sex groups as well as participants in same-sex groups (see Fig. 1). These results were partly in alignment with two previous studies, which also found that participants’ math test scores in same-sex groups were not affected by stereotype activation (Huguet & Regner, 2007; Inzlicht & Ben-Zeev, 2000). However, both studies found that stereotyped participants performed rather poorly in mixed-sex settings while non-stereotyped participants of the present study unexpectedly revealed generally enhanced cognitive performance. Thus, completing sex-sensitive cognitive tasks such as verbal fluency and perceptual speed in mixed-sex settings can increase cognitive performance as long as gender stereotypes are not activated. This partly explains why participants in mixed-sex groups and in the control condition generally outperformed their corresponding counterparts in same-sex groups and the gender stereotype condition, at least in verbal fluency and PS.

Although the effects of gender stereotypes on cognitive sex differences in different group sex settings seem relatively consistent across cognitive abilities, some tasks were found to be more sensitive to gender-stereotyping and/or group sex composition than others. Whether this observation is based on task difficulty and/or how strong and active participants’ gender stereotypes are in a given sample needs further investigation. From the present study, it is clear, however, that gender-stereotyping and group sex composition affect all three of the cognitive domains we tested.

### Arousal and Cognitive Sex Differences

The effects of gender-stereotyping and group sex composition can be interpreted within the framework of the Yerkes-Dodson law (Yerkes & Dodson, 1908), according to which there is an inverted U-shaped relationship between arousal and cognitive performance: low and high levels of arousal are associated with low cognitive performance while intermediate levels of arousal are related to high cognitive performance. In same-sex groups, whose gender stereotypes had not been activated, arousal was likely to be lower as compared to mixed-sex groups in which the competitive test situation might have increased arousal to an intermediate, beneficial level. This might explain why cognitive performance in same-sex groups was generally lower than in mixed-sex groups. Support for the notion that participants’ expectation of being compared with others might have increased performance in mixed-sex settings comes from a study by Niederle and Vesterlund (2007). In this study, participants in mixed-sex groups performed better on a simple math test when they believed their scores would be compared with each other, in contrast to when they believed their scores would not be compared. If in addition, however, gender stereotypes were activated in mixed-sex groups, arousal might have exceeded a critical threshold where it became detrimental to cognitive performance. The idea that heightened arousal, which has been frequently shown to interfere with more complex and difficult tasks (e.g., Davis & Harvey, 1992; Zajonc, 1965, 1969), may contribute to stereotype threat, has previously been proposed by O’Brien and Crandall (2003) who found stereotype threat only in a more difficult (math) test. This arousal-based explanation of stereotype threat might also explain the stereotype threat effects in only the more demanding verbal fluency task (i.e., 4W) found in men of the present study.

### Testosterone, Gender Stereotypes, and Group Sex Composition

As expected, saliva T levels were significantly higher in men than women. However, in contrast to Hypothesis 3 neither the stereotype threat men experienced in verbal fluency nor the increase in cognitive performance of non-stereotyped participants in mixed-sex groups was mediated by T. In addition, gender stereotype activation did not lead to changes in T levels and, in general (Hypothesis 4), T levels were not related to cognitive performance, despite the relatively large sample size of the present study.

This finding was in contrast to the prediction by Josephs et al. (2003) who suggest that participants with high baseline T levels are more susceptible to (gender-) stereotype threat than participants with low baseline T levels (see also Newman et al., 2005). We also did not find any support for the notion that an increase in T levels in gender-stereotyped men might underlie stereotype boost in mental rotation (particularly in mixed-sex groups) because male participants in all groups did not differ in T levels, and no gender stereotype effect was found in mental rotation.

### Implications for Co-Education

Proponents of single-sex education often argue that mixed-sex settings have detrimental effects, particularly on girls’ performance and school success in math and science (e.g., Sax, Arms, Woodruff, Riggers, & Eagan, 2009). The findings from Inzlicht and Ben-Zeev (2000) as well as Huguet and Regner (2007) seem to support these claims as they showed reduced math performance in females in the presence of males. The present findings, however, showed that mixed-sex settings can, in fact, boost cognitive performance—as long as gender stereotypes are not activated. And even if gender-stereotypes were activated, cognitive performance did not differ from same-sex groups. In a recent review, Halpern et al. (2011) came to the conclusion that there “is no well-designed research showing that single-sex (SS) education improves students’ academic performance” (p. 1706). Rather, by segregating males and females, single-sex education would strengthen gender stereotypes. The findings from the present study were consistent with Halpern et al. and may additionally suggest that given the right setting—with detrimental gender stereotype effects removed, for instance, by teaching about stereotype threat (Johns, Schmader, & Martens, 2005)—a mixed-sex, co-education environment might, in fact, be superior.

### Conclusion

Taken together, the present study showed that the cognitive performance of men and women was affected by gender stereotypes and group sex composition. First, the present study was one of the very few that found a stereotype threat in men’s cognitive performance (i.e., verbal fluency). Previous studies suggested that stereotype threat was more likely to affect men’s social and emotional intelligence (Koenig & Eagly, 2005; Leyens, Desert, Croizet, & Darcis, 2000). Second, the present study demonstrated that an interaction of gender stereotyping and group sex composition affected the performance of men and women in sex-sensitive cognitive tasks. Mixed-sex settings can, in fact, enhance performance in sex-sensitive cognitive tasks. This probably occurs when the test environment is appraised as challenging, thereby raising the arousal level close to its optimum. However, when gender stereotypes are additionally activated, the testing situation might be evaluated as threatening and performance is likely to be reduced. This is a strong argument against proponents of single-sex schooling who argue that mixed-sex settings have generally detrimental effects on performance. Finally, the present study did not find any interaction between gender-stereotyping and T levels: Gender-stereotyping neither affected T levels nor were baseline T levels related to the susceptibility to stereotype threat. In fact, the present study did not find any evidence for a relationship between baseline T and cognitive performance.
